# Global Transcriptome and Correlation Analysis Reveal Cultivar-Specific Molecular Signatures Associated with Fruit Development and Fatty Acid Determination in *Camellia oleifera* Abel

**DOI:** 10.1155/2020/6162802

**Published:** 2020-08-29

**Authors:** Shaofeng Peng, Jia Lu, Zhen Zhang, Li Ma, Caixia Liu, Yongzhong Chen

**Affiliations:** ^1^Beijing Forestry University, Haidian, 100083 Beijing, China; ^2^Hunan Academy of Forestry, Changsha, 410004 Hunan, China; ^3^National Engineering Research Center for Oil-Tea Camellia, Changsha, 410004 Hunan, China; ^4^Central South University of Forestry and Technology, Changsha, 410004 Hunan, China

## Abstract

**Background:**

Oil-tea Camellia is a very important edible oil plant widely distributed in southern China. Tea oil extracted from the oil-tea Camellia seeds is beneficial to health and is considered as a health edible oil. We attempt to identify genes related to fatty acid biosynthesis in an oil-tea Camellia seed kernel, generated a comprehensive transcriptome analysis of the seed kernel at different developmental stages, and explore optimal picking time of fruit. *Material and Methods*. A gas chromatography-mass spectrometer was used to detect the content of various fatty acids in samples. Transcriptome analysis was performed to detect gene dynamics and corresponding functions.

**Results:**

Multiple phenotypic data were counted in detail, including the oil content, oleic acid content, linoleic acid content, linolenic acid content, fruit weight, fruit height, fruit diameter, single seed weight, seed length, and seed width in different developmental stages, which indicate that a majority of indicators increased with the development of oil-tea Camellia. The transcriptomics was conducted to perform a comprehensive and system-level view on dynamic gene expression networks for different developmental stages. Short Time-series Expression Miner (STEM) analysis of XL106 (the 6 time points) and XL210 (8 time points) was performed to screen related fatty acid (FA) gene set, from which 1041 candidate genes related to FA were selected in XL106 and 202 related genes were screened in XL210 based on GO and KEGG enrichment. Then, candidate genes and trait dataset were combined to conduct correlation analysis, and 10 genes were found to be strongly connected with several key traits.

**Conclusions:**

The multiple phenotypic data revealed the dynamic law of changes during the picking stage. Transcriptomic analysis identified a large number of potential key regulatory factors that can control the oil content of dried kernels, oleic acid, linoleic acid, linolenic acid, fresh seed rate, and kernel-to-seed ratio, thereby providing a new insight into the molecular networks underlying the picking stage of oil-tea Camellia, which provides a theoretical basis for the optimal fruit picking point.

## 1. Introduction

Oil-tea Camellia (*Camellia oleifera* Abel.) is an ancient oilseed plant that has been cultivated for more than two thousand years and is mainly planted in tropical and subtropical regions [1]. Oil-tea Camellia is widely distributed in 11 provinces (municipality) in southern China, such as Hunan, Jiangxi, and Guangxi. Oil-tea Camellia has the characteristics of strong adaptability, high product value, and good comprehensive benefits. Owing to the high production of oils, oil-tea Camellia is considered one of the four woody oil plants, and the other three oil plants are oil palm, oil olive, and oil coconut. Tea oil is extracted from the oil-tea Camellia seeds and is widely used as an edible oil and a traditional medicine in our country. In particular, the Camellia oil is beneficial to health and is considered as a dietary oil [2]. Research indicated that tea oil contains abundant unsaturated FAs (fatty acids), including oleic acid, monounsaturated fatty acid, and linoleic acid [3]. Some reports systematically investigated the oil contents and fatty acid (FA) compositions of 11 wild oil-tea Camellia cultivars, revealing that oil contents in Camellia seeds varied from 41.92% to 53.30%. The average ratio and compositions of single FA were similar, and all involved palmitic acid (C16:0, PA), palmitoleic acid (C16:1), stearic acid (C18:0, SA), oleic acid (C18:1, OA), linoleic acid (C18:2, LA), linolenic acid (C18:3), eicosenoic acid (C20:1), and tetracosenoic acid (C24:1) [4].

Optimal picking time of fruit plays a pivotal role in the quality of the fruit. If the picking time is too early, the nutrients such as protein, sugar, and lipid in the fruit have not yet reached the optimal value. On the contrary, late harvest may lead to fruit aging and loss of nutrients. Hence, the optimal picking time of fruit of oil-tea Camellia can ensure the highest contents of FA. Usually, fruits of most cultivars are picked in October. However, the optimal picking time is still unknown. Therefore, 6 time points and 8 time points were separately selected in two common cultivars (XL106 and XL210) to systemically explore the indicator value of characters, including weight of single fruit, fruit width, seed number of fruit, seed length, seed thick, kernel weight, linolenic acid, oleic acid, and palmitic acid. Furthermore, changes in gene expression were analyzed by using transcriptomics.

On the one hand, we aimed to explore whether the oil content increases or decreases when fruits were picked in the early or late stages. If the difference is not significant, we can pick the fruit at any time from September to November, which is conducive to guiding the production. On the other hand, we attempt to identify genes related to the FA biosynthesis in oil-tea Camellia seeds, generated a comprehensive transcriptome of the seed kernel at different developmental stages, and explored the molecular regulatory mechanisms.

## 2. Materials and Methods

### 2.1. Plant Materials

The experimental materials of XL210 and XL106 were obtained from the Germplasm Resources Preservation Base of Hunan Academy of Forestry (National Oil-Tea Engineering Technology Research Center), which is located in Mingyue Village, Lukou Town, Changsha, Hunan Province, China (N28° 24.29, E113° 15.19′). XL106 is an excellent clone by the Hunan Academy of Forestry Sciences in 1994. The flowering stage is from early October to mid-late December. The fruit maturity stage is in early October, 25-60 fruits per 500 grams. The yield of fresh seed is 47.7%, and seed kernel oil content is 42.95%. XL210 is a good strain of seeds in 2003 by the Hunan Academy of Forestry Sciences. It has a white flowering period from late October to late December. The fruit ripening stage is in late October, 15-30 fruits per 500 grams. The fresh seed rate is 44.8%, and the seed kernel oil rate is 44.32%. XL210 and XL106 are planted and preserved in the germplasm resource garden of oil-tea Camellia in the Hunan Academy of Forestry Sciences.

The sampling time of XL210 is from September 15, 2018, to November 5, 2018, sampling every other week, a total of 8 samples. From September 15, 2018, to October 22, 2018, the XL106 was sampled every other week for a total of 6 samples. At each sample, 10 fruits were collected from the middle and upper parts of the outer canopy of the tree. After picking, it was immediately put into self-sealing bags and put into dry iceboxes. When they were taken back to the laboratory, the peel and seed coat were stripped off, and the seed kernel was taken out and stored at -80°C for later use. In total, 8 samples in XL210 and 6 samples in XL106 without biological duplication were obtained for subsequent transcriptome. Details are as follows: we find a beaker with a diameter slightly larger than the fruit of Camellia oil and fill it with clear water. We tie the fruit with a string and let the fruit fully immerse in water. Then, the overflow was caught with a measuring cylinder to measure the volume that is the volume of the fruit.

### 2.2. Analysis of Phenotypic Data

Ten fruits were randomly selected at each time, and the fruit weight, fruit volume, fruit height, fruit diameter, pericarp thickness, and ventricular number per seed were determined. Fruit height, fruit diameter, and peel thickness were determined by a vernier caliper. The fruit volume was measured by the drainage method.

About one g seed kernel of oil-tea Camellia was randomly collected in each time period. Firstly, the wall was broken mechanically, and then, the FA was extracted with the Lipid Extraction Kit. Then, the fatty acid in the seed kernel was determined in accordance with *GB 5009.168-2016 food safety national standard for methyl ester treatment*.

### 2.3. Total RNA Isolation, Library Construction, and Transcriptome Sequencing

As mentioned earlier, RNA isolation and purification as well as cDNA library construction and sequencing were conducted [5]. In brief, total RNA was extracted from the seed kernel from both oil-tea Camellia cultivars using the TRIzol Reagent (Invitrogen, Beijing, China). The NanoDrop ND1000 spectrophotometer (NanoDrop Technologies, Wilmington, DE, USA) and the Agilent Bioanalyzer 2100 system (Agilent Technologies, Palo Alto, CA, USA) were used to determine the quantity and quality of RNA, respectively, and 1% agarose gel electrophoresis was used to detect RNA integrity. The mRNA was isolated from total RNA using magnetic beads with oligo (dT). The cDNA synthesis kit (TaKaRa) was selected to synthesize cDNA and linked the sequencing adapter at both ends of cDNA [6]. All RNA-Seq libraries were sequenced on a NovaSeq, and the whole set of the transcriptome raw data have been deposited in the Sequence Read Archive (SRA) database of the National Center for Biotechnology Information (NCBI) (accession number SRP241856).

### 2.4. De Novo Transcriptome Assembly and Analysis of Differentially Expressed Genes (DEGs)

For further analysis, the adaptor reads and low-quality reads were removed to obtain the clean reads, and then, the trinity platform (http://trinityrnaseq.sourceforge.net/) was used for transcriptome assembly without a reference genome. The unigene sequences obtained were integrated for annotation.

For gene expression analysis, the counts were mapped to the reading of each gene by HTSeq v0.5.4p3 and then normalized to fragments per kilobase of transcript per million mapped reads (FPKM) according to the report [7]. DEGs were screened following log2 (fold change) ≥ 1 and corrected *P* ≤ 0.005. GOseq (1.10.0) [8] and KOBAS software [9] were utilized to conduct Gene Ontology (GO) enrichment and Kyoto Encyclopedia of Genes and Genomes (KEGG) enrichment for all DEGs. The time series analysis software Mfuzz was used to perform the clustering algorithm [10]. Mfuzz is a software package that can classify genes into multiple clusters based on similar expression profiles, which helps find genes with similar functions. At the same time, genes with the same expression trend may participate in the same biological process, which helps to find gene regulation.

### 2.5. Real-Time PCR Analysis

Real-time PCR was performed according to a previous report [11]. Briefly, the PrimeScript RT Kit (TaKaRa, Dalian, China) was used to reverse-transcribe RNA into cDNA according to the manufacturer's instructions. A 20 *μ*L scale was selected to conduct the PCR reaction using the SYBR Premix ExTaq™ (TaKaRa, Dalian, China) on an ABI (Applied Biosystems) StepOne Plus System. The relative expression of target genes was calculated by the 2^−*ΔΔ*Ct^ method, and 12 correlated genes were selected for RT-qPCR using specific primers designed by Primer Premier 5 software. Each measurement was carried out in triplicate, and data is expressed as means ± standard error (SE).

## 3. Results

### 3.1. Indicator Value of Characters of Two Oil-Tea Camellia Cultivars in the Developmental Stages

**Figure 1:**
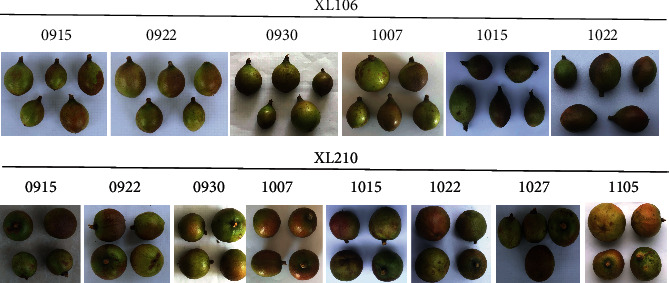
Fruit morphology of the XL106 and XL210 in different developmental stages.

From the appearance of the fruit, it has no significant difference between the two cultivars at different stages of development (Figure 1). Indicator values in different periods of XL106 and XL210 are detailedly described in Tables 1 and 2. Results indicated that a majority of indicators keep increasing with growth of fruit. In particular, the oil content of dried kernels is significantly increased during the developmental stages, XL106 increased from 37.70% to 64.51%, and XL210 increased from 32.09% to 59.92%. Many characters have a larger difference between the two cultivars. XL106 matures early, with small flowers, small leaves, and small fruits, but the oil content is relatively high. However, XL210 matures later, with larger flowers, leaves, and fruits and low oil content. Throughout the development process, the palmitic acid content of XL106 was higher than that of XL210. The average content of oleic acid of XL106 was 75.69%, which was higher than XL210. Simultaneously, other related traits of fruit were systematically measured including fruit weight, fruit height, fruit diameter, single seed weight, seed length, and seed width, which is relatively higher in XL210.

### 3.2. RNA-Seq Analysis

In order to explore the molecular basis of the above-described morphological differences in seed development, RNA-Seq analyses were conducted to generate transcriptome profiles. A total of 11.52 million clean reads from the samples were screened following a series of data filtering. The proportion of the GC accounts for 49% (Supplementary Table 1), and the percentage of reads with an average quality score of more than 30 was more than 94% (Supplementary Table 1), indicating that the sequencing data was accurate, quality, and sufficient for further analysis. 174310 unigenes were assembled by using trinity, with an average length of 591.16 bp (Supplementary Table 2). The median length of contig (N50) was 816 bp, and the range of 69097 unigenes is from 200 to 300 bp (Supplementary Table 3).

### 3.3. Differential Gene Expression during Seed Kernel Development in Two Cultivars

**Figure 2:**
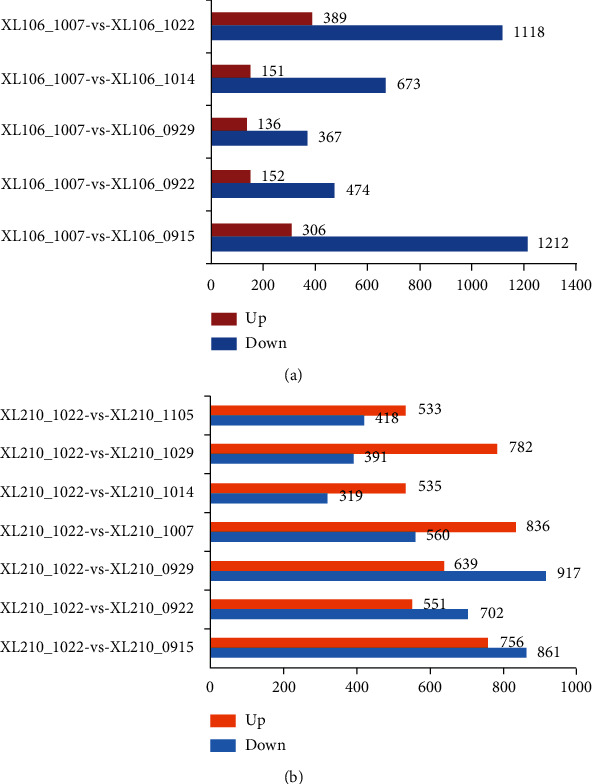
Analysis of the number of differential genes at different time points. (a) Analysis of the number of differential genes of XL106 at different time points. (b) Analysis of the number of differential genes of XL210 at different time points.

The transcriptome analysis of different stages of seed kernel development can provide crucial system-level insights into the molecular mechanisms underlying seed development. By convention, the picking time of XL106 and XL210 is selected on October 7 and October 22, respectively, where the oil content is usually the highest. Therefore, this time point with other groups was compared, and the corresponding results are exhibited in Figure 2. In the XL106 group, compared with XL106_1007, the number of DEG in the initial stage (XL106_0915 with 1518 DEG) and the mature stage (XL106_1022 with 1507 DEG) is higher than other developmental stages (including XL106_0922 with 626, XL106_0930 with 503DEG, and XL106_1014 with 824 DEG), in which the number of downregulated genes is more than that of the number of upregulated genes (Figure 2(a)).

Among the eight stages of XL210, 1617 DEGs and 1253 DEGs were detected in the early stage (XL210_0915 and XL210_0922), in which the number of upregulated genes is lower than that of downregulated genes. 1396 and 854 DEGs separately were screened in the middle developmental stage (XL210_1007 and XL210_1014), in which the number of upregulated genes was more than that of downregulated genes. At seed maturity (XL210_1029 and XL210_1105), 1173 and 951 DEGs are identified, and the number of upregulated genes is more than that of the downregulated genes (Figure 2(b)).

### 3.4. KEGG Analysis of Differential Genes

**Figure 3:**
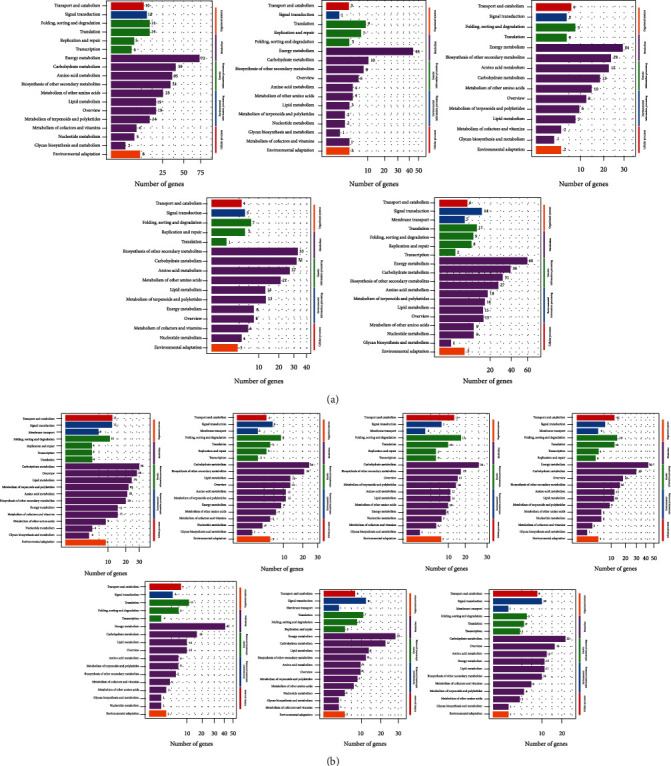
KEGG classification of differentially expressed genes. (a) KEGG classification analysis of DEGs in XL106 (from left to right: XL106_1007 vs. XL106_0915, XL106_1007 vs. XL106_0922, XL106_1007 vs. XL106_0930, XL106_1007 vs. XL106_1014, and XL106_1007 vs. XL106_1022). (b) KEGG classification analysis of DEGs in XL210 (from left to right: XL210_1022 vs. XL210_0915, XL210_1022 vs. XL210_0922, XL210_1022 vs. XL210_0930, XL210_1022 vs. XL210_1007, XL210_1022 vs. XL210_1014, XL210_1022 vs. XL210_1029, and XL210_1022 vs. XL210_1105).

KEGG pathway analysis was conducted to explore the function of DEGs at different stages of two cultivars. The results of XL106 showed that the enriched pathway of KEGG in the initial period (XL106_1007 vs. XL106_0915) and mature period (XL106_1007 vs. XL106_1022) was roughly similar and mainly enriched in the pathway, including energy metabolism, amino acid metabolism, FA metabolism, carbohydrate metabolism, and biosynthesis of other secondary metabolites. These pathways are positively related to the FA metabolism of oil-tea Camellia (Figure 3(a)). However, compared with the initial period, the pathway of membrane transport was significantly enriched in the mature period. For the XL210, although the initial and mature stages (XL210_1022 vs. XL210_0915 and XL210_1022 vs. XL210_1105) were enriched in pathways similar to XL106, the number of genes in the pathways varied greatly, respectively, reducing nearly half of the gene (Figure 3(b)).

### 3.5. Dynamic Changes of DEGs in Different Developmental Processes of Two Cultivars

**Figure 4:**
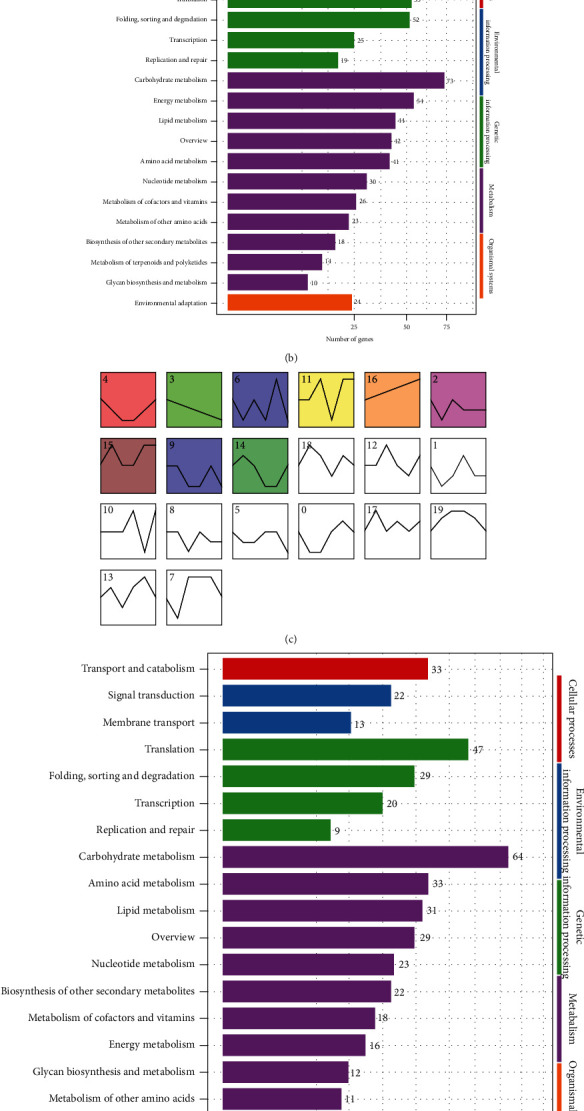
Trend analysis of gene expression (10 trends) and corresponding KEGG classification of gene. KEGG classification of cluster 9 (a) and KEGG classification of cluster 16 (b) and trend analysis of gene expression in XL106 (c). KEGG classification of cluster 0 (d). Trend analysis of gene expression in XL210 (e). Colored block trend: significant enrichment trend. Without color trend: the enrichment of significant trends.

Based on STEM time series analysis, trend analysis was performed on XL106 (the 6 time points) (Figure 4(a)) and XL210 (8 time points) (Figure 4(b)) to screen related trend gene sets. All of the genes were clustered into 26 profiles, of which nine trend profiles were significantly enriched (*P* < 0.05) (colored block). Through continuously observing the fatty acid content, we more focus on the gene set: continuously increased gene sets and gene set of “down-up-down.” In XL106, the trend gene set of No.9 and No.16 was screened, and the analysis of KEGG enrichment was conducted. The results suggested that many genes annotated to translation (62 genes), overview (58 genes), and carbohydrate metabolism (55 genes) in gene set of No.9. The No.16 gene set indicated that 73 genes annotated carbohydrate metabolism, 54 genes for energy metabolism, and 53 genes for translation. Similarly, we more focus on cluster 0 in the XL210, indicating that carbohydrate metabolism, translation, amino acid metabolism, and fatty acid metabolism were the mainly annotated pathways.

### 3.6. Correlation Analysis between Fatty Acid-Related Genes and Phenotype Indicates the Differential Regulatory Network of Two Cultivars

**Figure 5:**
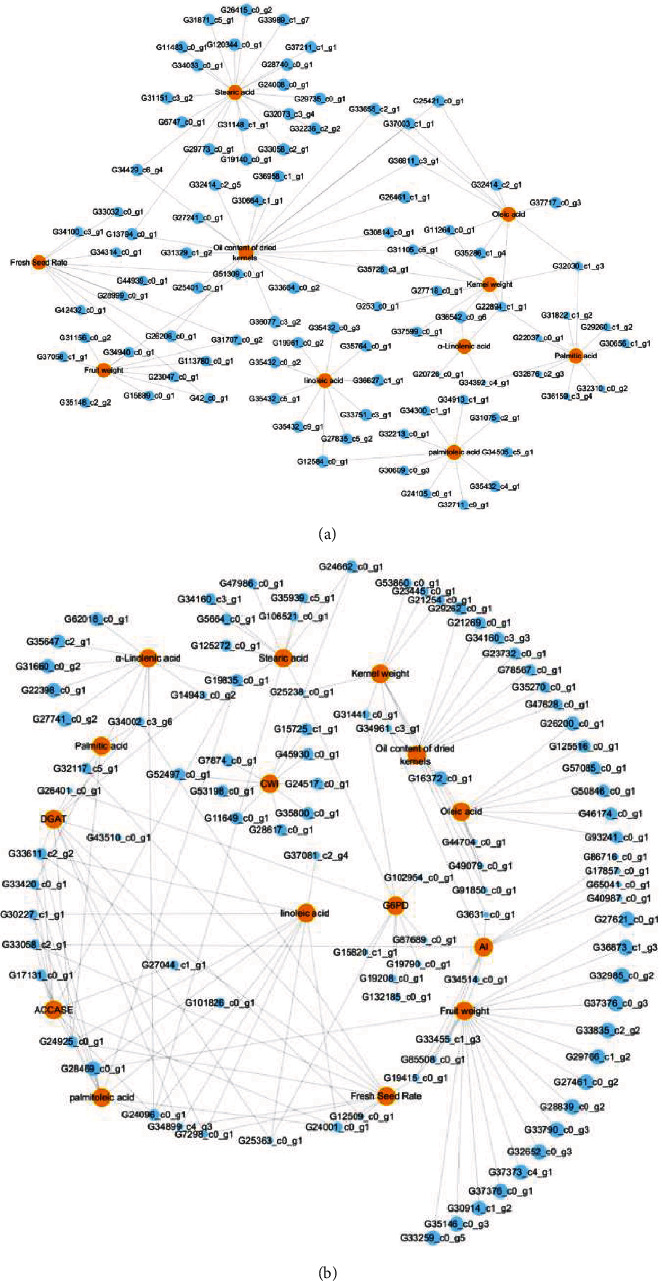
Correlation network diagram of gene and character. Red nodes represent character, and blue nodes represent gene. (a) Character-gene network of XL106; (b) character-gene network of XL210.

Firstly, the FA-related genes were screened from the top 20 data of GO and KEGG pathway (cluster 9 and cluster 16 in XL106 and cluster 0 in XL210), including 1041 candidate genes related to fatty acid was selected in the XL106 group (Supplementary Table 4) and 202 related genes were screened in the XL210 group (Supplementary Table 5). Subsequently, candidate genes and trait dataset were combined to conduct correlation analysis (Figure 5). We further obtained a pair of strong correlations between genes and traits and finally screened the top 10 genes with positive correlations for each trait. The connection between genes and traits represents a strong correlation, and the genes connecting multiple traits were the key genes.

In the trait-gene correlation network diagram of XL106, a number of genes with strong correlations with different physiological indicators were found including G37003_c1_g1 (DnaJ protein), G33655_c2_g1 (glycerol-3-phosphate acyltransferase), G36811_c3_g1 (lipid transfer protein), G22894_c1_g1 (chlorophyll a-b binding protein), G37717_c0_g3 (respiratory burst oxidase homolog protein), G35286_c1_g4 (mannitol dehydrogenase), G_3241_g3 (uncharacterized protein), and G3241_g2 (uncharacterized protein), which have a strong connection with several key traits such as the oil content of dried kernels, oleic acid, linoleic acid, linolenic acid, fresh seed rate, and kernel-to-seed ratio.

Similarly, in XL210, many genes were found, including G29262_c0_g1, G34899_c4_g3 (scarecrow-like protein), G26200_c0_g1 (uncharacterized protein), G7298_c0_g1 (polygalacturonase At1g48100), G34002_c3_g6 (sucrose synthase), and G34160_c3_g3 (uncharacterized protein). And the genes have a strong connection with dried kernel oil content, oleic acid, linoleic acid, linolenic acid, fresh seed rate, and kernel-seed ratio. Specific related genes and molecular functions are also listed in Table 3.

### 3.7. Specific Genes Involved in the Production of Particular Fatty Acids and the Regulation and Synthesis of Neutral Lipid or Triacylglycerol

**Figure 6:**
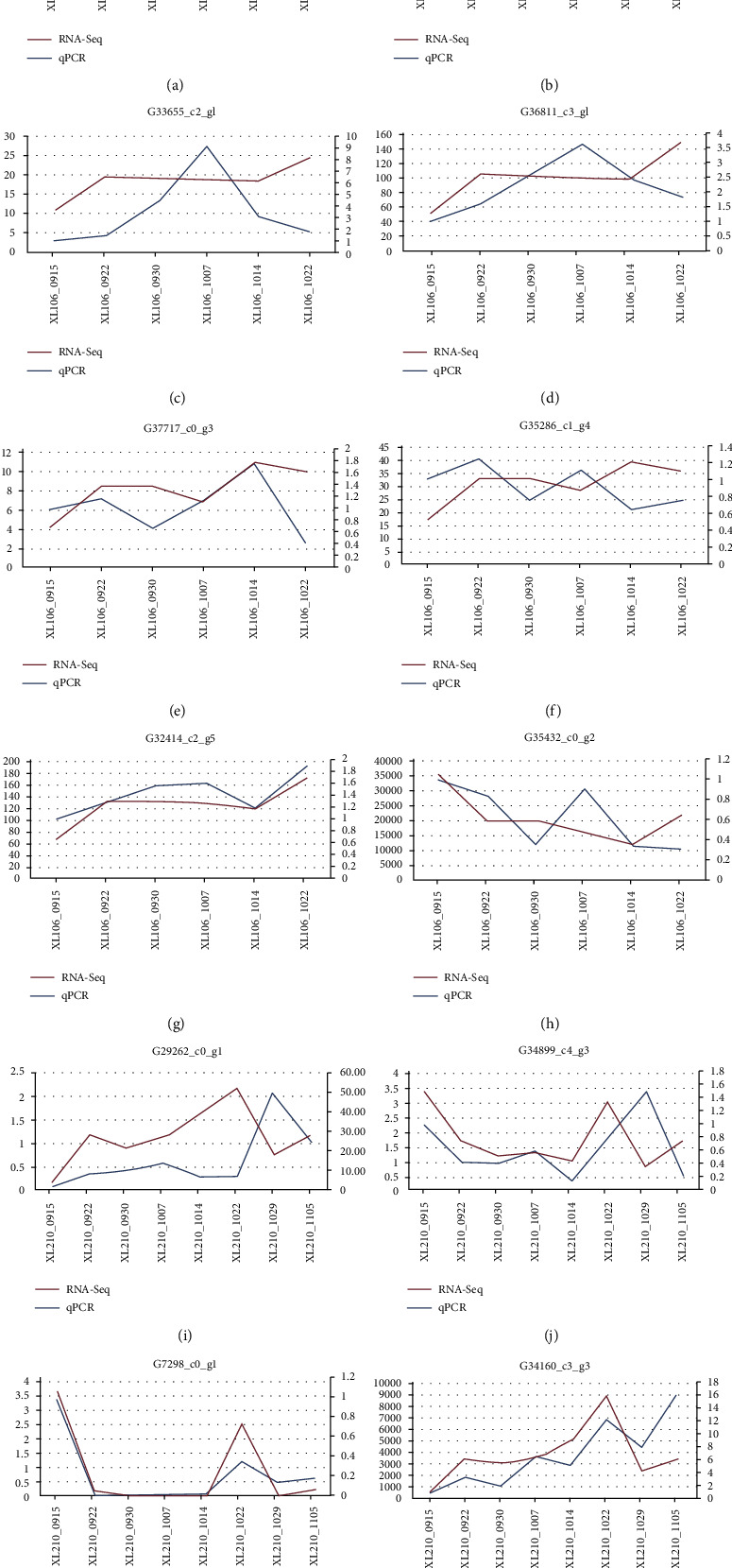
The expression of representative genes in XL210 and XL106 validated by qRT-PCR.

Linoleic acid and linolenic acid are essential fatty acids for the human body and must be supplied by food. Thus, we focused on the genes related to synthesis. As shown in Figure 6, we screened the genes related to linoleic acid including G35432_c0_g3 (globulin seed storage protein), G35764_c0_g1 (protein SCAR3), G36627_c1_g1 (polyadenylate-binding protein), g33751_c3_g1 (actin-related protein), G27835_c5_g2 (heterogeneous nuclear ribonucleoprotein), G12584_c0_g1 (calmodulin-like protein), G35432_c9_g1 (globulin seed storage protein), G35432_c5_g1 (globulin seed storage protein), G35432_c0_g2 (globulin seed storage protein), and G19961_c0_g2 (uncharacterized protein). G37599_c0_g1 (pleiotropic drug resistance protein), G36542_c0_g6 (ribonucleotide reductase large subunit B family protein), G22894_c1_g1 (chlorophyll a-b binding protein 13), G34392_c4_g1 (hypothetical protein), and G20726_c0_g1 (histone H2A-like) were found to be related to linolenic acid.

### 3.8. RT-qPCR Validation of the Transcriptomic Data

Finally, 12 genes related to FA (8 genes in XL106 and 4 genes in XL210) were screened, and the expression levels in different developmental stages were analyzed using RT-qPCR. The results indicated that the majority of 8 genes in XL106 had similar expression patterns between the RNA-Seq results and RT-qPCR (Figure 6). Most of the genes showed a continuous increase over time and maintained a relatively stable state.

In XL210, the expression line graph showed that the expression trend of 4 genes at eight time points was almost identical, indicating that the expression quantity increased gradually with time and fluctuates at the last two time points. The results validated the good consistency of the RNA-Seq data and RT-qPCR gene expression.

## 4. Discussion

Understanding the molecular regulation mechanisms of FA is of great importance to instruct the production and FA yield in oil-tea Camellia. In this study, transcriptomic comparison was performed between several developmental stages of XL106 and XL210 seed kernel. The results provided comprehensive information about genes related to the determination of FA.

According to the changes of FA and combined current meteorological conditions, two cultivars were selected for systematic analysis. Usually, XL106 was picked around October 7, and XL210 was picked on October 22; thus, a few key points before and after picking were chosen. We have studied the phenotypic traits and studied the different developmental stages of two cultivars in detail, demonstrating that most indicators maintain growth over time. However, many indicators, including palmitic acid, stearic acid, oleic acid, linoleic acid, and linolenic acid, showed a trend of “down-up-down.” With the development of plants and the maturation of fruit, fatty acid, protein, fat, and other nutrients continue to increase. The phenomenon is commonplace and has been reported in many plants, especially in oil crops including peanut [12] and rapeseed [13]. However, related FAs have not continued to increase during the stages of the time period of picking (September to October). In particular, compared with the conventional picking point, the content of FA including palmitic acid, stearic acid, oleic acid, linoleic acid, and linolenic acid was gradually decreased in the later period, speculating that fruits are starting to senescence after the optimal picking period and fatty acids start to decompose and consume [14].

Therefore, transcriptome analysis was performed and the molecular regulatory mechanisms were explored. As shown in Supplementary Table 1, the average data volume of each sample reached 2.3 G, and the proportion of base quality reaching Q30 is 94%, which is better than other researches, indicating that quality of sequencing is normal and satisfied the following analysis [15, 16]. DEG analysis showed that the number and variation of upregulated and downregulated genes of the two cultivars at different stages have great changes. The differential regulation of gene members in different cultivars may lead to different regulatory networks, which can determine the fruit development and FA content of specific varieties.

KEGG analysis showed that the results of XL106_1007 vs. XL106_0915 (early stage) and XL106_1007 vs. XL106_1022 (later stage) were similar and were mainly enriched in the pathway including energy metabolism, amino acid metabolism, fatty acid metabolism, carbohydrate metabolism, and biosynthesis of other secondary metabolites. These massively concentrated pathways have a great relationship with the synthesis of oil-tea Camellia [17, 18]. In the two stages, the number of KEGG-enriched genes was 385 and 346, respectively, which indicated that a large number of the genes and pathways related to FA remained active in the pre- and poststage of XL106. A large number of genes and pathways remain active. Compared with XL106_1007 vs. XL106_0915, it is worth mentioning that the enrichment results of XL106_1007 vs. XL106_1022 found that there was a distinct and significant enrichment in the metabolic pathways related to membrane transport. The results may mean that more pathways are needed to perform more transmembrane transport that adapted to accommodate the large accumulation of FA at the later stages [19, 20]. Aiming at XL210, the enrichment pathways were found to be similar to XL106, but the initial and later stage numbers of genes on the pathways have greatly changed, 292 and 152 genes, respectively, which means that XL210 was delayed by 2 periods at the time point. It was speculated that the long-term accumulation of fatty acid in the later period could negatively inhibit the activity of relevant metabolic pathways, thereby maintaining the normal growth and metabolic balance of the organism [21].

STEM time series analysis was carried out, and the trend gene set related to the targeted expectation was found according to 6 time points of XL106 and 8 time points of XL210. It was also found that several genes were strongly correlated with different physiological indicators at the same time. In particular, many genes with a strong correlation with the character were screened, including dry kernel oil content, oleic acid, linoleic acid, linolenic acid, fresh seed ratio, and kernel-to-seed ratio.

G33655_c2_g1 (glycerol-3-phosphate acyltransferase) is a kind of acyltransferase, which can play a key role in the metabolic pathway of glycerophosphofatty acid [22, 23]. Previous studies have found that glycerol-3-phosphate acyltransferase played essential roles in the production of triacylglycerol in Phaeodactylum tricornutum [24, 25]. G37717_c0_g3 gene is NADPH oxidase, which can activate or respond to the MAPK signal transduction pathway [26], thus responding to endogenous or exogenous stimuli including growth factors [27], cytokines, radiation, and osmotic pressure [28]. It is speculated that it can respond to the flow of metabolic products, such as oleic acid. G35286_c1_g4 (mannitol dehydrogenase) and G34002_c3_g6 (sucrose synthase) participate in the regulation of nutrients or energy metabolism and fatty acid metabolism by affecting ammonium phenylpropionate metabolism, endoplasmic reticulum protein processing, and starch and sugar metabolism pathways [29].

In the synthesis process of oil-tea Camellia fatty acid metabolites and other metabolites, it will be affected by the regulation of a lot of genes, including signal transduction, energy metabolism, the product of base material synthesis, and key synthetase gene functions [30–32]. These different levels of gene interaction formed a complex biomolecular regulatory network, which in turn affects the synthesis, accumulation, and metabolism of fatty acid metabolites [33, 34]. Overall, on the one hand, we revealed the dynamic change of various FAs. On the other hand, the analyses of the comprehensive transcriptome dataset in this study provided a useful genomic resource for fruit picking and provided molecular insights into the related FA genes.

## Figures and Tables

**Table 1:** Indicator value of characters in different periods of XL106.

Trait indicators	Sample collection date					
	9-15	9-22	9-29	10-7	10-14	10-22
Palmitic acid (%)	10.976	11.789	11.664	11.327	11.165	11.217
Palmitoleic acid (%)	0.148	0	0.099	0	0	0
Stearic acid (%)	0.89	1.13	1.632	1.523	1.667	2.529
Oleic acid (%)	66.516	76.839	76.394	77.154	79.058	78.149
Linoleic acid (%)	21.272	10.034	9.709	9.472	8.109	8.105
Linolenic acid (%)	0.198	0.208	0.502	0.514	0	0
Oil content of dried kernels (%)	37.70	49.93	55.25	57.16	62.28	64.51
Single fruit weight (g)	8.8224	9.7412	11.4632	7.7862	8.296	11.6192
Fruit volume (cm^3^)	7.04	9.1	9.96	6.68	7.64	9.96
Fruit height (mm)	29.388	30.308	31.324	28.06	28.32	30.422
Fruit diameter (mm)	23.834	24.468	26.404	22.886	22.936	26.384
Peel thickness (mm)	2.838	2.598	2.86	2.86	2.756	3.308
Seeds per fruit (pcs)	1.6	1.8	1.8	1.2	1.2	2.2
Fresh seed weight (g)	4.1362	4.5178	5.6566	3.5942	3.6704	5.7838
Fresh seed rate (%)	46.88	46.38	49.35	46.16	44.24	49.78
Single seed weight (g)	2.6474	2.7524	3.1792	3.0516	3.1058	2.6524
Seed volume (cm^3^)	2.12	2	2.38	1.96	2.32	2.14
Seed length (mm)	17.688	18.412	19.872	16.854	19.092	18.152
Seed width (mm)	18.598	18.98	20.982	19.326	17.736	18.428
Seed thickness (mm)	15.436	13.308	12.36	15.904	15.88	12.366
Seed coat thickness (mm)	0.826	0.712	0.746	0.734	0.77	0.756
Kernel-seed ratio	0.66	0.69	0.68	0.71	0.70	0.72

**Table 2:** Indicator value of characters in different periods of “XL210”.

Trait indicators	Different sampling periods							
	9-15	9-22	9-29	10-7	10-14	10-22	10-29	11-5
Palmitic acid (%)	16.22	15.35	16.40	16.65	14.78	14.34	16.72	13.39
Palmitoleic acid (%)	0.185	0.168	0.232	0.182	0.178	0.16	0	0
Stearic acid (%)	1.075	1.02	0.91	1.228	2.379	1.063	0.758	1.233
Oleic acid (%)	65.07	71.50	63.14	66.88	71.05	70.45	71.90	76.52
Linoleic acid (%)	17.02	11.77	19.17	14.85	11.24	13.65	10.47	8.75
Linolenic acid (%)	0.43	0.21	0.15	0.21	0.37	0.34	0.15	0.11
Oil content of dried kernels (%)	32.09	59.34	43.57	46.52	55.33	63.64	61.13	59.92
Single fruit weight (g)	32.04	35.94	32.62	31.00	31.58	40.02	31.40	32.62
Fruit volume (cm^3^)	30.75	35.75	32	30.25	30.25	39	31.13	32.5
Fruit height (mm)	33.43	34.71	36.41	35.18	35.42	37.61	35.10	34.48
Fruit diameter (mm)	40.27	39.91	39.23	38.05	37.21	41.36	39.24	38.82
Peel thickness (mm)	4.12	5.08	4.23	4.36	4.44	5.09	4.53	4.32
Seeds per fruit (pcs)	6	6.25	5	4.25	5.5	7.25	4	4.5
Fresh seed weight (g)	14.23	13.94	13.73	12.28	11.44	15.97	12.70	9.46
Fresh seed rate (%)	44.42	38.78	42.08	39.61	36.23	39.89	40.45	28.99
Single seed weight (g)	2.1978	2.637	3.1166	3.2258	2.3396	2.4246	3.0932	2.633
Seed volume (cm^3^)	1.76	1.96	2.6	2.4	1.6	1.8	2.24	2.2
Seed length (mm)	17.172	16.472	20.368	18.092	15.452	18.448	16.28	17.37
Seed width (mm)	19.02	18.096	19.946	20.976	20.618	17.884	21.552	19.604
Seed thickness (mm)	12.994	13.17	14.516	16.616	11.728	13.182	17.352	14.16
Seed coat thickness (mm)	0.758	0.832	0.788	0.688	0.612	0.6	0.712	0.738
Kernel-seed ratio	0.6210	0.6538	0.6781	0.6763	0.6731	0.6787	0.6911	0.6848
DGAT (U/g)	0.2531	0.1794	0.2128	0.2543	0.1865	0.1720	0.1815	0.1874
AI (U/g)	4768.00	8757.17	7772.19	6450.67	5235.86	6746.17	7419.23	5391.82
G6PD (U/g)	79.3842	65.1789	77.7872	86.5289	61.3124	64.0862	59.8835	78.2915
CWI (U/g)	1180.62	1361.16	2244.74	1675.51	2038.71	2329.7	1291.06	1471.60
ACCase (U/g)	0.4317	0.3994	0.4204	0.4060	0.3837	0.3738	0.2950	0.2523

DGAT: diacylglycerol acyltransferase; AI: invertase; G6PD: glucose-6-phosphatase dehydrogenase; CWI: cell wall-bound invertase; ACCase: acetyl coenzyme A carboxylase.

**Table 3:** Target genes and related descriptions.

Gene ID	NR database ID	NR database notes	KO notes	Metabolic pathway
G37003_c1_g1	OVA02738.1	DnaJ domain (Macleaya cordata)		
G33655_c2_g1	XP_017231148.1	Predicted: probable glycerol-3-phosphate acyltransferase 8 (Daucus carota)	Glycerol-3-phosphate acyltransferase	Glycerophospholipid metabolism
G36811_c3_g1	CAN67019.1	Hypothetical protein VITISV_027707 (Vitis vinifera)		
G22894_c1_g1	XP_002284493.1	Predicted: chlorophyll a-b binding protein 13, chloroplastic (Vitis vinifera)	Light-harvesting complex II chlorophyll a/b binding protein 3	Photosynthesis—antenna proteins
G37717_c0_g3	AII25876.1	NADPH oxidase A (Camellia sinensis)	Respiratory burst oxidase	MAPK signaling pathway
G35286_c1_g4	XP_010095885.1	Putative mannitol dehydrogenase (Morus notabilis)	Cinnamyl-alcohol dehydrogenase	Phenylpropanoid biosynthesis
G32414_c2_g5	XP_007010484.1	Predicted: dnaJ protein homolog (Theobroma cacao)	DnaJ homolog subfamily A member 2	Protein processing in endoplasmic reticulum
G35432_c0_g2	NP_001291336.1	11S globulin seed storage protein 2 precursor (Sesamum indicum)		
G29262_c0_g1	XP_011100902.1	Alkane hydroxylase MAH1-like (Sesamum indicum)		
G34899_c4_g3	XP_015062026.1	Predicted: scarecrow-like protein 22 (Solanum pennellii)		
G26200_c0_g1	CDO98741.1	Unnamed protein product (Coffea canephora)		
G7298_c0_g1	XP_002266600.1	Predicted: polygalacturonase At1g48100 (Vitis vinifera)		
G34002_c3_g6	AHL29281.1	Sucrose synthase 1 (Camellia sinensis)	Sucrose synthase	Starch and sucrose metabolism
G34160_c3_g3	XP_011075467.2	Kirola-like (Sesamum indicum)		

## Data Availability

The datasets used and/or analyzed during the current study are available from the corresponding author on reasonable request.

## Supplementary Materials

Supplementary Materials Supplementary Table 1: transcriptome base statistics after filtration. Supplementary Table 2: the length range of transcript and unigene. Supplementary Table 3: the length of transcript, unigene, and N50. Supplementary Table 4: candidate genes related to fatty acid in XL106. Supplementary Table 5: candidate genes related to fatty acid in 202.
