# Supplementary material for: Global Transcriptome and Correlation Analysis Reveal Cultivar-Specific Molecular Signatures Associated with Fruit Development and Fatty Acid Determination in Camellia oleifera Abel
Source: Int J Genomics. 2020 Aug 29;2020:6162802. doi: 10.1155/2020/6162802 (PMC7481963; doi:10.1155/2020/6162802)
Supplement: Supplementary Materials — Supplementary Table 1: transcriptome base statistics after filtration. Supplementary Table 2: the length range of transcript and unigene. Supplementary Table 3: the length of transcript, unigene, and N50. Supplementary Table 4: candidate genes related to fatty acid in XL106. Supplementary Table 5: candidate genes related to fatty acid in 202. [file 6162802.f1.zip › Supplementary table 1.docx]

Supplementary table 1 Transcriptome base statistics after filtration

| Numbers of samples | Numbers of Clean Reads | Base(M) | Q20 rate | Q30 rate | GC rate |
| --- | --- | --- | --- | --- | --- |
| XL106_0915 | 71197226 | 10606 | 98.25 | 94.86 | 47.69 |
| XL106_0922 | 91328332 | 13596 | 98.43 | 95.28 | 47.97 |
| XL106_0929 | 85514660 | 12760 | 98.2 | 94.69 | 47.98 |
| XL106_1007 | 79572430 | 11873 | 96.65 | 91 | 50.84 |
| XL106_1014 | 84597728 | 12604 | 97.12 | 91.94 | 47.22 |
| XL106_1022 | 80315610 | 11949 | 98.32 | 95.02 | 47.27 |
| XL210_0915 | 73253362 | 10890 | 98.51 | 95.49 | 48.71 |
| XL210_0922 | 69601986 | 10382 | 98.41 | 95.22 | 47.01 |
| XL210_0929 | 83701414 | 12463 | 98.24 | 94.94 | 48.48 |
| XL210_1007 | 84172968 | 12548 | 97.14 | 92.05 | 49.82 |
| XL210_1014 | 73969694 | 11026 | 96.75 | 91.31 | 50.26 |
| XL210_1022 | 75342858 | 11225 | 98.33 | 95.1 | 47.32 |
| XL210_1029 | 125440122 | 18699 | 97.77 | 93.78 | 53.54 |
| XL210_1105 | 74424560 | 11087 | 98.32 | 95.08 | 50.6 |
| Average | 82316639.29 | 12264.857 | 97.88857 | 93.98286 | 48.90786 |
